# Insights on the Evolution of Prolyl 3-Hydroxylation Sites from Comparative Analysis of Chicken and Xenopus Fibrillar Collagens

**DOI:** 10.1371/journal.pone.0019336

**Published:** 2011-05-03

**Authors:** David M. Hudson, MaryAnn Weis, David R. Eyre

**Affiliations:** Department of Orthopaedics and Sports Medicine, University of Washington, Seattle, Washington, United States of America; University of Queensland, Australia

## Abstract

Recessive mutations that prevent 3-hydroxyproline formation in type I collagen have been shown to cause forms of osteogenesis imperfecta. In mammals, all A-clade collagen chains with a GPP sequence at the A1 site (P986), except α1(III), have 3Hyp at residue P986. Available avian, amphibian and reptilian type III collagen sequences from the genomic database (Ensembl) all differ in sequence motif from mammals at the A1 site. This suggests a potential evolutionary distinction in prolyl 3-hydroxylation between mammals and earlier vertebrates. Using peptide mass spectrometry, we confirmed that this 3Hyp site is fully occupied in α1(III) from an amphibian, *Xenopus laevis*, as it is in chicken. A thorough characterization of all predicted 3Hyp sites in collagen types I, II, III and V from chicken and xenopus revealed further differences in the pattern of occupancy of the A3 site (P707). In mammals only α2(I) and α2(V) chains had any 3Hyp at the A3 site, whereas in chicken all α-chains except α1(III) had A3 at least partially 3-hydroxylated. The A3 site was also partially 3-hydroxylated in xenopus α1(I). Minor differences in covalent cross-linking between chicken, xenopus and mammal type I and III collagens were also found as a potential index of evolving functional differences. The function of 3Hyp is still unknown but observed differences in site occupancy during vertebrate evolution are likely to give important clues.

## Introduction

Fibrillar collagens are found throughout all metazoan phyla. In fact, collagen is the most abundant animal protein, with 28 or more different collagen types produced by over 40 genes in vertebrates [1], [2]. Collagens undergo many post-translational modifications, such as the conversion of approximately 50% of all prolyl residues to (4*R*, 2*S*-L-hydroxyproline; 4Hyp), which make up approximately a quarter of the amino acid content of most fibrillar α-chains [1]. Similarly, 25% or more of lysine residues are hydroxylated to 5-hydroxylysine, some of which are glycosylated. It is well established that these post-translational modifications enhance collagen structural stability [3], [4]. However, the function of the much less frequent 3-hydroxylation of proline in the collagen α1(I) chain to form 3-hydroxyproline (3*S*, 2*S*-L-hydroxyproline; 3Hyp) is unclear [5].

Interest has focused on 3Hyp recently, with the discovery that gene mutations causing recessively inherited forms of osteogenesis imperfecta also can block 3Hyp formation at the P986 site in collagen α1(I) [6]–[12]. It was shown that the protein complex responsible for P986 3Hyp formation is made up of prolyl 3-hydroxylase, cartilage associated protein (CRTAP) and cyclophylin B [13], [14], and that mutations in the genes encoding any one of these proteins can result in recessive osteogenesis imperfecta [6]–[12]. The significance of the lack of P986 3-hydroxylation in the pathology is uncertain, however.

Clearly, 3Hyp is an ancient and ubiquitous collagen modification, found throughout as far back as porifera [15]. It seems unlikely that a post-translational modification as rare but conserved as 3Hyp would not contribute basically to collagen structure and function. At which point in evolution 3-hydroxylation first appeared in fibrillar collagens, and specifically at P986, is unknown. A single prolyl 3-hydroxylase gene is present in the genome of the pre-vertebrate chordate, *Ciona intestinalis*; genomic duplications resulted in three prolyl 3-hydroxylases in higher vertebrates [16].

We recently revealed several previously unknown molecular sites of 3Hyp along fibrillar collagen chains using peptide mass spectrometry [17]. Both A-clade chains (α1(I), α2(I), α1(II), α1(III) and α2(V)) and B-clade chains (α1(V), α1(XI) and α2(XI)) were examined from human and bovine tissues. Four A-clade sites (A1, A2, A3 and A4) and three B-clade sites (B1, B2 and B3) were defined. A particularly interesting finding was 100% 3-hydroxylation of the A1 site in type III collagen from chicken skin. It was predicted that the presence of a histidine six residues N-terminal to the A1 proline prevented 3-hydroxylation of mammalian type III collagen [17]. The available avian, amphibian and reptilian sequences from the genomic database (Ensembl) all contained a tyrosine, not histidine, in the sequence motif, suggesting potential 3-hydroxylation at the A1 site of type III collagen of earlier vertebrates in contrast to mammals. To further understand potential evolutionary changes in the occurrence of 3Hyp in vertebrates, we examined the known 3Hyp sites in collagen type I, II, III and V chains from xenopus and chicken by tandem mass spectrometry.

## Materials and Methods

### Collagen extraction and fractionation

Unless otherwise indicated, all steps were carried out at 4°C. Based on established protocols [18], types I, III and V collagens were isolated from the skin of chicken wings purchased at the supermarket and *Xenopus laevis* purchased from Xenopus Express, Inc. (Brooksville, FL). Briefly, acid soluble collagen was extracted from defatted (chloroform: methanol, 3∶1 v/v) skin with 0.5 M acetic acid for 24 hours. The pellet was digested with 0.2 mg/mL pepsin in 0.5 M acetic acid for 24 hours before the addition of pepstatin (7 µg/ml). Type I and type III collagens were precipitated at 0.85 M NaCl and resolubilized in 50 mM Tris-Cl, pH 7.4, 1 M NaCl for 24 hours. After centrifugation most of the type III collagen was recovered in the insoluble fraction. The small amount of type III collagen that remained in the supernatant was not further fractionated. Type V collagen was precipitated at 1.2 M NaCl from the pepsin digest. Types I, III and V collagens were individually dialyzed against 0.1 M acetic acid and lyophilized. Extraction and fractionation of xenopus skin collagen required the addition of *o*-phenanthroline (5 mM) to extractants, which prevented partial degradation of collagen α-chains seen on electrophoresis. Type II collagen was isolated from the cartilage of both species as previously described [17]. Protein samples were analyzed by sodium dodecyl sulphate polyacrylamide gel electrophoresis (SDS-PAGE) and stained with Coomassie Blue G-250 (Sigma-Aldrich) [19].

### Mass spectrometry

Selected collagen α-chains resolved by SDS polyacrylamide electrophoresis were excised and subjected to in-gel trypsin digestion [20]. Peptides were analyzed by electrospray mass spectrometric analysis as previously described [17]. Samples were analyzed using an LCQ Deca XP ion-trap mass spectrometer equipped with in-line liquid chromatography (LC) (ThermoFinnigan) using C8 capillary column (300 µm×150 mm; Grace Vydac 208MS5.315). An electrospray ionization source introduced the LC sample stream into the mass spectrometer. Sequest search software (ThermoFinnigan) was used for peptide identification searching the NCBI protein database and the Ensembl genome database. Larger collagenous peptides were not found by Sequest and had to be identified manually by calculating the possible MS/MS ions and matching these to the actual MS/MS spectrum. Hydroxyl differences were searched for manually by scrolling or averaging the full scan over several minutes so that all the post-translational variations of a given peptide were combined in the full scan.

Protein sequences used for MS analysis were obtained from the NCBI protein and Ensembl genome databases (genomic sequences were always used when available). The following sequences were used for chicken: col3a1: (Ensembl entry: ENSGALG00000002552), col1a1: (NCBI Reference Sequence: P02457.3), col1a2: (Ensembl entry: ENSGALG00000009641), col2a1: (Ensembl entry: ENSGALG00000013587), col5a1: (Ensembl entry: ENSGALG00000002546), and col5a2: (Ensembl entry: ENSGALG00000002509). The following sequences were used for xenopus: col3a1: (NCBI Reference Sequence: NP_001083544.1), col1a1: (NCBI Reference Sequence: NP_001080821.1), col1a2: (NCBI Reference Sequence: NP_001080727.1), and col2a1: (NCBI Reference Sequence: NP_001081258.1).

### Characterization of collagen cross-links

The pyridinoline cross-link contents of collagen type I and III preparations were determined by HPLC after acid hydrolysis in 6 N HCl for 24 hours at 108°C. Dried samples were dissolved in 1% (v/v) n-heptafluorobutyric acid and analyzed by reverse-phase HPLC detecting hydroxylysyl pyridinoline and lysyl pyridinoline by fluorescence monitoring as previously described [21].

## Results

### Isolation of collagen α-chains

**Figure 1:**
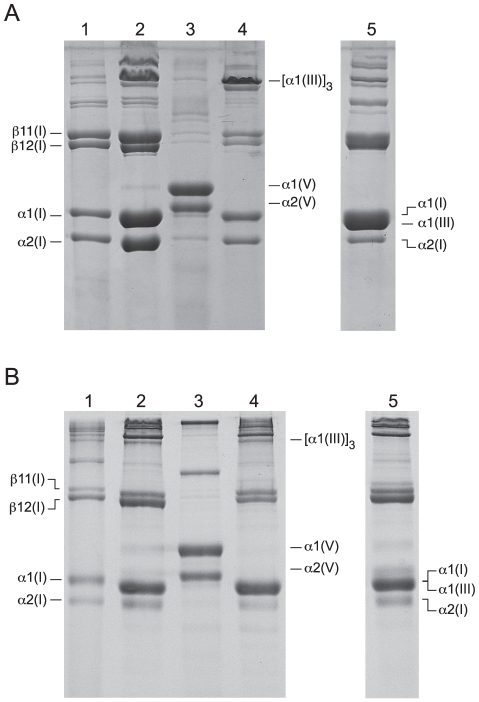
Isolated collagen species from chicken and xenopus on 6% SDS-PAGE. A, chicken collagens; lane 1, acid soluble fraction; lane 2, neutral 1.0 M salt soluble fraction ; lane 3, acid 1.2 M salt fraction; lane 4, neutral salt insoluble fraction; lane 5, reduced neutral salt insoluble fraction. B, xenopus collagens; lane 1, acid soluble fraction; lane 2, neutral 1.0 M salt soluble fraction; lane 3, acid 1.2 M salt fraction; lane 4, neutral salt insoluble fraction; lane 5, reduced neutral salt insoluble fraction. β11(I) and β12(I) are cross-linked α1-α1 and α1-α2 chain dimers, respectively.

Isolation of the major collagens from chicken and xenopus skin was based on an established purification protocol [18]. Dilute acetic acid extracted 20% of the type I collagen from both chicken and xenopus skin preparations. Type III and V collagens were solubilized only on pepsin treatment together with the bulk of the remaining type I collagen. Chicken dermis types I, III, and V constituted approximately 78%, 20% and 2% of the total collagen, based on SDS-PAGE and dry weight of salt precipitated fractions (Figure 1A). The type III collagen preparation was easily identified on SDS-PAGE comparing runs with and without disulfide bond reduction (Figure 1A; lanes 4 and 5). Collagen types I, III and V from xenopus skin made up about 90%, 5% and 5% of the total isolated collagen (Figure 1B). Neutral-salt extraction of the 0.85 M NaCl collagen precipitate failed to separate xenopus types I and III; however, a small amount of trimeric type III collagen was identified in both the soluble and insoluble neutral salt preparations by running ±DTT on SDS-PAGE (Figure 1B; lanes 2, 4 and 5).

### Substrate consensus sequence of the 3Hyp primary site

Collagen α-chains α1(I), α2(I), α1(II), α1(III), α1(V) and α2(V), were screened for the presence of 3Hyp using mass spectrometry, as described previously [17]. In-gel trypsin digests were inspected for the presence of mass variants (+16 Da) at the known molecular sites of 3Hyp (A1, A2, A3 and A4) for the A-clade collagens and (B1, B2 and B3) for the B-clade collagens.

**Figure 2:**
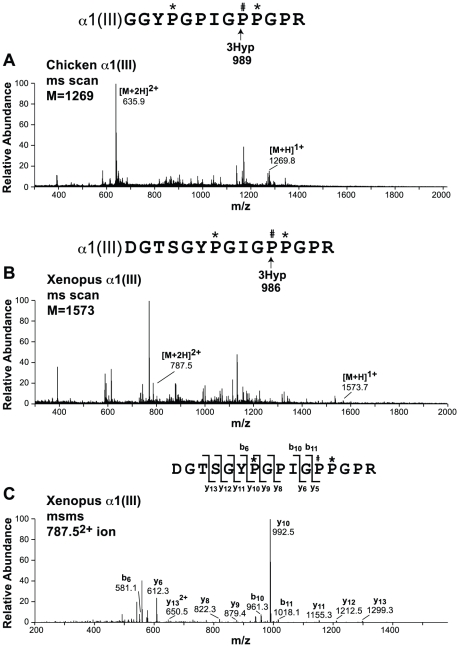
Mass spectra of tryptic peptides containing the A1 site from chicken and xenopus α1(III). Comparison of full scan spectra from LC-MS profiles of in-gel trypsin digests of the homologous sequence in the α1(III) chain from A, chicken; and B, xenopus. C, MS/MS analysis of the 3Hyp containing tryptic peptide from xenopus.

The A1 site was ≥99% hydroxylated in all the α-chains tested from xenopus and chicken skin, except α2(I), which lacks a GPP motif at this locus. Mass spectrometry confirmed that this 3-hydroxy modification was indeed 100% occupied in the α1(III) chain of xenopus, as it is in chicken (Figure 2). This was particularly interesting since all mammalian species tested lacked 3Hyp at the A1 site of α1(III) [17]. We concluded that 3Hyp formation at the A1 site in tested mammals was blocked by local sequence differences. Sequence comparison between human (GTSG**H**PGPIGPPGPR), chicken (GRGG**Y**PGPIGPPGPR) and xenopus (GTSG**Y**PGPIGPPGPR) reveals the only notable local difference between mammalian and non-mammalian sequences is His to Tyr at position 980 (983 in chicken).

### An uncharacteristic pattern of 3Hyp sites in chicken collagen

**Figure 3:**
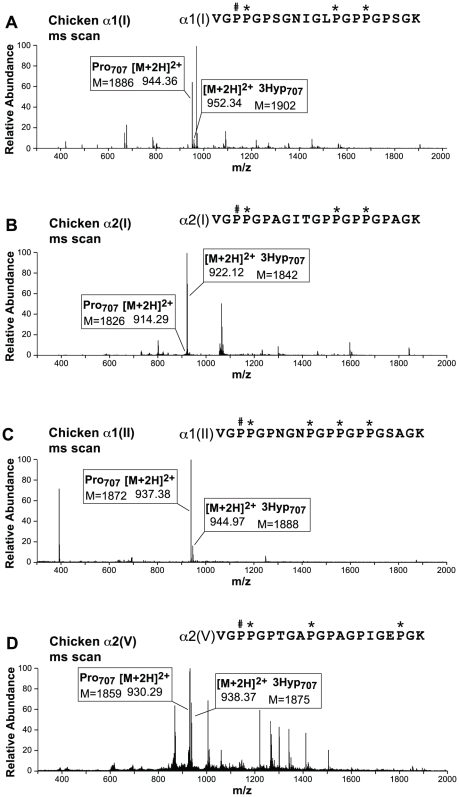
Mass spectra of tryptic peptides containing the A3 site in chicken fibrillar collagens. Full scan spectra from LC-MS profiles of in-gel trypsin digests of α1(I) from chicken bone, A; α2(I) from chicken skin, B; α1(II) from chicken cartilage, C; and α2(V) from chicken skin, D. The degree of hydroxylation at P707 is estimated through a correlation with the relative abundance of the representative ions. P*, 4Hyp; P^#^, 3Hyp.

The A3 site (P707) in chicken showed a distinct pattern of 3-hydroxylation from mammals. It was previously shown that only α2(I) and α2(V) chains were 3-hydroxylated at the A3 site in mammals, each with up to 80% occupancy [17]. In chicken, the A3 site of the α2(I) and α2(V) chains was 3-hydroxylated (95% and 40%, respectively), but in addition the A3 site of chicken α1(I) and α1(II) was also occupied (10% and 18%, respectively) (Figure 3), whereas in mammals it was not. To determine if this was sample or tissue related, we also isolated and examined chicken α1(I) from bone and tendon and found a similar occupancy at P707 for all three tissues. In fact, of the A-clade α-chains only α1(III) lacked hydroxylation at the A3 site. Analysis of the predicted tryptic peptide sequence for the α1(I) A3 site revealed either an error in the chicken genomic database or a chicken strain variance, with an Ala to Ser substitution at residue 723, which gave a misleading +16 mass, the site of which was clarified using MS/MS. Similar to chicken, the xenopus A3 site (P707) of α1(I) is 12% 3-hydroxylated (data not shown). Of particular interest, the A3 site of xenopus α2(I) has no GPP sequence. In contrast, human bone α1(I) P707 was not hydroxylated at all, but α2(I) P707 was 80% 3-hydroxylated [17].

**Figure 4:**
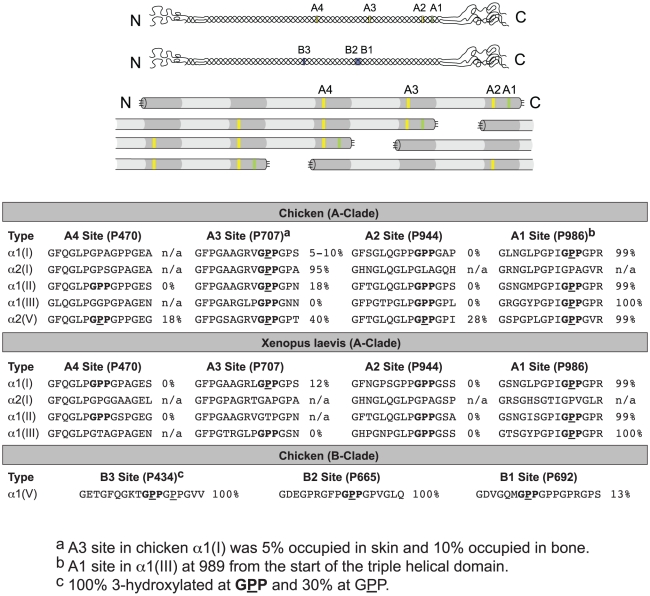
Chicken and xenopus sequences and locations of 3Hyp residues in A-clade and B-clade collagen α-chains. D-periodic spacing is evident between the 3Hyp residues at sites A4 and A3 and between sites A3 and A2 in the A-clade triple helical procollagen molecule; and between sites B3 and B2 in the B-clade triple helical procollagen molecule. GPP sequences containing potential 3Hyp sites are shown in bold with occupied sites underlined. Percentage of 3Hyp occupancy relative to the unmodified sequence is indicated. Absence of the GPP sequence is indicated with n/a.

As in mammals, chicken α2(V) was the only A-clade α-chain that had all four 3Hyp sites hydroxylated, with occupancies of 99%, 28%, 40% and 18% at sites A1, A2, A3 and A4, respectively. The candidate 3Hyp molecular sites in the chicken B-clade collagen α1(V) chain were more hydroxylated than in mammals with the B3 and B2 sites each 100% occupied. Interestingly, the B3 site had a second GPP site C-terminally in series (GPPGPP) with occupancy of 30%. The α2(V) and α1(V) chains of xenopus were not analyzed for 3Hyp as there is currently no reliable sequence information for *Xenopus laevis* type V collagen genes in any publicly available database. The results are summarized in Figure 4.

### Cross-linking properties of type III collagen in chicken

The unique presence of 3Hyp in chicken type III collagen prompted a search for possible distinct functional properties. Potential differences in covalent cross-linking were sought as an index of an altered polymeric form. Collagen types I and III from chicken skin gave low yet measurable levels of hydroxylysyl pyridinoline, at 0.04 and 0.09 mole/mole of collagen, respectively. Collagens isolated from mammalian skin typically give undetectable levels of hydroxylysyl pyridinoline and lysyl pyridinoline by fluorescence HPLC [22], so the hydroxyallysine pathway contributes at a low level to cross-linking of chicken skin but not mammalian skin.

## Discussion

The present results show several dissimilarities in 3Hyp occupancy between mammalian and lower vertebrate A-clade and B-clade collagen chains. For example, the A3 site is partially 3-hydroxylated in xenopus α1(I) and all chicken A-clade α-chains except α1(III). Evolutionarily, it seems that amphibians and birds have retained the modification at P707, while mammals either have lost it or never gained it ancestrally. It is significant that xenopus lacks the prerequisite GPP sequence at the A3 site of α2(I) that is conserved in mammals (Ensembl entry: ENSG00000164692), birds (Ensembl entry: ENSTGUP00000001601), the anole lizard (Ensembl entry: ENSACAG00000013301) and even the zebrafish (Ensembl entry: ENSDARP00000042989). It is important to note however, that the A3 site of zebrafish α2(I) is completely unoccupied by 3Hyp (Eyre and Weis unpublished results). Moreover, none of the other available fish sequences from the Ensembl genomic database contain a GPP motif at the A3 site. It is therefore tempting to speculate that the A3 site emerged with the tetrapods, as a possible gain of function modification. It appears that under selective pressure, the 3Hyp A3 site has predominantly localized to the α2(I) and α2(V) chains of higher vertebrates.

Another major difference between mammals and chicken and xenopus, is the 100% occupancy of the α1(III) A1 site in chicken and xenopus. Type III collagen appears to have branched off from a common ancestor earlier than the other A-clade collagens [23], which could suggest a functional divergence in α1(III) between phyla. It has been established that 3Hyp, compared to Pro, in the *X* position of the tripeptide Gly-*X*-4Hyp results in a slight increase in the melting temperature (*T_m_*) of the triple-helical peptide [24]. Indeed, chickens have a core body temperature that ranges from 39–45°C, several degrees higher than that of mammals [25], and xenopus, a poikilotherm, has evolved to adapt to a wide range of external temperatures [26]. Yet an inspection of 4Hyp content in chicken reveals 136 residues per α1(III) chain [18] and 111 residues per α1(I) chain [27], whereas humans have 124 residues per α1(III) chain [28] and 101 residues per α1(I) chain [29]. It seems unlikely that 1 or 2 extra 3Hyp residues evolved solely to contribute an additional thermal stability to the molecule as a whole.

There is evidence that chicken type III collagen has unique physical and biochemical properties. Early studies examining the substrate specificity of human skin fibroblast collagenase (MMP1) showed marked differences in the enzyme catalytic rates with different collagen substrates *in vitro*. Human type III collagen was cleaved 10× faster than human type I collagen [30]. Yet chicken type III was cleaved at one hundredth the rate of mammalian type III [31]. No significant difference in collagenase activity was observed between chicken and mammalian type I collagen. One possible explanation is that occupancy of 3Hyp at position P989 in chicken α1(III) promotes lateral association of molecules in solution that bestows greater resistance to proteolytic digestion of the relatively unstable triple-helical domain that contains the Gly775 Leu/Ile collagenase cleavage site compared with monomeric molecules in solution. We recently proposed a role for 3Hyp in promoting lateral associations in fibril assembly [17].

It should also be considered that type III collagen in chicken may form thick fibrils in its own right suggesting a more prominent role for this collagen in birds. Type III collagen is typically found in vertebrates co-distributed with type I collagen predominantly in cyclically extensible connective tissues such as skin, blood vessels, uterus and other internal organs [32]. Although it is nominally a fibril-forming collagen in mammals, it appears to polymerize only in the form of fine microfibrils covalently linked to the surface of the much thicker fibrils of copolymeric collagen, as observed for example with type II/XI fibrils in cartilage [33]. We suspect, therefore that 3Hyp plays a role in the macromolecular assembly of collagen fibrils, perhaps by aiding or fine tuning the D-periodic staggered alignment of molecules in the initial assembly of collagen fibrils through intermolecular hydrogen bonding (illustrated in Figure 4). As such, the exposed hydroxyls could mediate bonds between triple-helices, including pairs of collagen molecules in register [17]. This concept is consistent with molecular interactions required to explain the chemistry and location of cross-links in fibrils. Fibrillar collagens are typically heavily cross-linked, high tensile-strength, thick fibrils. The present cross-linking analyses showed low levels of hydroxylysyl pyridinoline in chicken skin types I and III collagens. This is consistent with the formation of homopolymeric type III collagen fibrils. 3Hyp in chicken type III collagen may therefore enhance its capacity for fibril formation compared to mammalian type III collagen. Though high levels of type III collagen were not observed in our xenopus skin preparations, differences in extractability and/or protease susceptibility of type III collagen between chicken and xenopus may explain the apparent poor yield from xenopus.

The complete lack of prolyl 3-hydroxylation at the A1 site of mammalian type III collagen has potentially interesting implications in vertebrate evolution. As yet any functional significance for 3Hyp remains speculative. However, the evidence of selective pressures on 3Hyp site occupancy implies a functional role for the modification itself rather than being simply a coincidental mark of the hydroxylase complex acting as a chaperone during assembly and transport of collagen in the ER. We speculate that 3Hyp formation impacted the mechanism of collagen fibril assembly at the threshold of vertebrate evolution in a way that benefited the development and diversification of connective tissues.
